# Calculated Plasma Volume Status Is Associated with Adverse Outcomes in Patients Undergoing Transcatheter Aortic Valve Implantation

**DOI:** 10.3390/jcm10153333

**Published:** 2021-07-28

**Authors:** Hatim Seoudy, Mohammed Saad, Mostafa Salem, Kassem Allouch, Johanne Frank, Thomas Puehler, Mohamed Salem, Georg Lutter, Christian Kuhn, Derk Frank

**Affiliations:** 1 Department of Internal Medicine III, Cardiology and Angiology, Campus Kiel, University Hospital Schleswig-Holstein, D-24105 Kiel, Germany; hatim.seoudy@uksh.de (H.S.); mohammed.saad@uksh.de (M.S.); mostafa.salem@uksh.de (M.S.); kassem.allouch@gmx.de (K.A.); johanne.frank@uksh.de (J.F.); chris_kuhn@gmx.de (C.K.); 2 DZHK (German Centre for Cardiovascular Research), Partner Site Hamburg/Kiel/Lübeck, D-24105 Kiel, Germany; thomas.puehler@uksh.de (T.P.); mohamed.salem@uksh.de (M.S.); georg.lutter@uksh.de (G.L.); 3 Department of Cardiac and Vascular Surgery, Campus Kiel, University Hospital Schleswig-Holstein, D-24105 Kiel, Germany

**Keywords:** aortic stenosis, transcatheter aortic valve implantation, valvular heart disease, congestion, plasma volume, risk stratification

## Abstract

Background: Calculated plasma volume status (PVS) reflects volume overload based on the deviation of the estimated plasma volume (ePV) from the ideal plasma volume (iPV). Calculated PVS is associated with prognosis in the context of heart failure. This single-center study investigated the prognostic impact of PVS in patients undergoing transcatheter aortic valve implantation (TAVI). Methods: A total of 859 TAVI patients had been prospectively enrolled in an observational study and were included in the analysis. An optimal cutoff for PVS of −5.4% was determined by receiver operating characteristic curve analysis. The primary endpoint was a composite of all-cause mortality or heart failure hospitalization within 1 year after TAVI. Results: A total of 324 patients had a PVS < −5.4% (no congestion), while 535 patients showed a PVS ≥ −5.4% (congestion). The primary endpoint occurred more frequently in patients with a PVS ≥ −5.4% compared to patients with PVS < −5.4% (22.6% vs. 13.0%, *p* < 0.001). After multivariable adjustment, PVS was confirmed as a significant predictor of the primary endpoint (HR 1.53, 95% CI 1.05–2.22, *p* = 0.026). Conclusions: Elevated PVS, as a marker of subclinical congestion, is significantly associated with all-cause mortality and heart failure hospitalization within 1 year after TAVI.

## 1. Introduction

Transcatheter aortic valve implantation (TAVI) has become an essential treatment option for severe aortic stenosis (AS) across the whole spectrum of surgical risk [1,2]. In patients undergoing TAVI, subclinical congestion is associated with worse clinical outcomes. However, it is often not detected by routine clinical assessment [3]. While right heart catheterization is considered the gold standard to quantify fluid status in patients with volume overload, its use is limited by its invasive nature [4]. Radiotracer indicator-dilution methods using labeled albumin or red blood cells were previously proposed as alternative techniques to accurately quantify plasma volume (PV), but are not applicable in daily clinical practice [5,6]. In contrast, non-invasive PV calculations based on weight and hematocrit have been shown to correlate well with quantitative measurements using gold-standard radioisotope assays [7]. Plasma volume status (PVS) reflects the degree of deviation of the estimated plasma volume (ePV) from the ideal plasma volume (iPV) and has been shown to be associated with prognosis in patients with heart failure [8].

The aim of this study was to determine the incidence and prognostic impact of subclinical volume overload, detected by elevated calculated PVS, in patients undergoing TAVI. We hypothesized that PVS is significantly associated with adverse outcomes after TAVI.

## 2. Materials and Methods

Between February 2014 and February 2020, a total of 979 patients undergoing transfemoral TAVI were prospectively enrolled in an observational study at the University Hospital Schleswig-Holstein, Kiel, Germany. Patients with incomplete clinical or follow-up data as well as those with intraprocedural conversion to open-heart surgery were excluded from the analysis. The final study population comprised 859 patients.

An optimal PVS cutoff value of −5.4% (AUC 0.601, 95% confidence interval 0.55–0.65, *p* < 0.001) was determined using receiver operating characteristic (ROC) curve analysis. According to calculated PVS, patients were divided into two groups: PVS < −5.4% (no congestion) versus PVS ≥ −5.4% (congestion). The primary endpoint was a composite of all-cause mortality or heart failure hospitalization. The secondary study endpoints were both individual components of the primary endpoint.

The decision to perform TAVI was based on careful evaluation by the multidisciplinary heart team. All procedures were performed using third-generation SAPIEN devices (Edwards Lifesciences, Irvine, CA, USA) or CoreValve devices (Medtronic, Minneapolis, MN, USA). Type and size of the transcatheter heart valve were determined using pre-procedural multidetector computed tomography measurements and evaluation with the 3 mensio Structural Heart software (3 mensio Medical Imaging BV, Bilthoven, the Netherlands). Procedures were done by experienced implanters and pre- and post-dilatation were left to the physician’s discretion. During TAVI, unfractionated heparin was administered to achieve an activated clotting time of 250–300 s. Closure of the vascular access was conducted using two Perclose ProGlide™ vascular closure systems (Abbott Laboratories, Chicago, IL, USA).

PVS was calculated after obtaining ePV and iPV using two well-established equations which have been previously reported in detail elsewhere [9,10]:

ePV = ((1 − hematocrit) × (a + (b × weight (kg)))), with a = 864 in females and 1530 in males, and b = 47.9 in females and 41 in males.

iPV = k × weight [kg], with k = 40 in females and 39 in males.

PVS is an index of deviation of ePV from iPV and was subsequently calculated as follows:

PVS = ((ePV − iPV) × 100%)/iPV

Written informed consent was obtained from each patient. The study was approved by the Ethics Committee of the University of Kiel and conformed to the ethical guidelines of the Declaration of Helsinki. Patient data and blood samples were collected 1–3 days prior to TAVI. Patients were followed up through clinical visits, communication with ambulatory physicians, or telephone consultations after the procedure. Patient outcomes were reported according to the incidence of life-threatening bleeding, myocardial infarction, stroke with disability, acute kidney injury stage 3/4, and new permanent pacemaker implantation in accordance with the definitions of the Valve Academic Research Consortium-3 (VARC-3) consensus document [11]. Hospitalization for heart failure was defined as hospitalization due to typical symptoms and objective signs of worsening heart failure. The Society of Thoracic Surgeons (STS) risk score was calculated using the updated model released in 2018 [12,13].

All continuous data showed a skewed distribution and were thus expressed as median with interquartile range (IQR). Categorical variables were summarized as counts and percentages. Differences between both groups were assessed using the Mann-Whitney U test, χ^2^-test, and Fisher’s exact test, as appropriate. Outcome data were evaluated using Kaplan-Meier curves and the log-rank test. For the Cox regression model, all factors linked to mortality in univariable (*p*-value < 0.25) were considered as candidate variables. The backward selection was based on the likelihood ratio criteria. Continuous variables were dichotomized to keep the Cox model simple. Cox regression results were presented as adjusted hazard ratios (HR) with 95% confidence intervals (CI). The proportional hazard assumption was confirmed using weighted residuals. In order to minimize collinearity, covariables directly related to PVS including hemoglobin, hematocrit, and anemia were not included in the regression model. Statistical analyses were performed using R software, version 4.0.4 (URL: https://www.R-project.org/ (accessed on 27 July 2021)), and GraphPad PRISM, version 8 (GraphPad Software, San Diego, CA, USA). All tests were two-tailed, and a *p*-value < 0.05 was considered statistically significant.

## 3. Results

### 3.1. Baseline Characteristics

A total of 859 TAVI patients were available for analysis. Based on the calculated PVS cutoff, 324 patients (37.7 %) had a PVS < −5.4% (no congestion), while 535 patients (62.3%) had a PVS ≥ −5.4% (congestion). Compared to patients with PVS < −5.4%, the PVS ≥ −5.4% group was significantly older, had a higher proportion of female patients as well as higher STS Scores, higher levels of NT-proBNP, higher rates of impaired left ventricular ejection fraction (LVEF), higher prevalence of anemia, lower eGFR, lower hematocrit and hemoglobin, lower BMI and lower prevalence of dyslipidemia. Diuretics were more frequently prescribed in patients with a PVS ≥ −5.4% compared to the PVS < −5.4% group (Table 1).

### 3.2. Periprocedural Complications

There was no statistically significant difference regarding the type of transcatheter heart valve used for the procedure. Periprocedural complications defined as individual endpoints of type 3 (life-threatening) bleeding, myocardial infarction, stroke with disability, acute kidney injury stage 3/4, and new pacemaker implantation after TAVI did also not differ between both groups (Table 2).

### 3.3. Clinical Outcome during Long-Term Follow-Up

**Figure 1:**
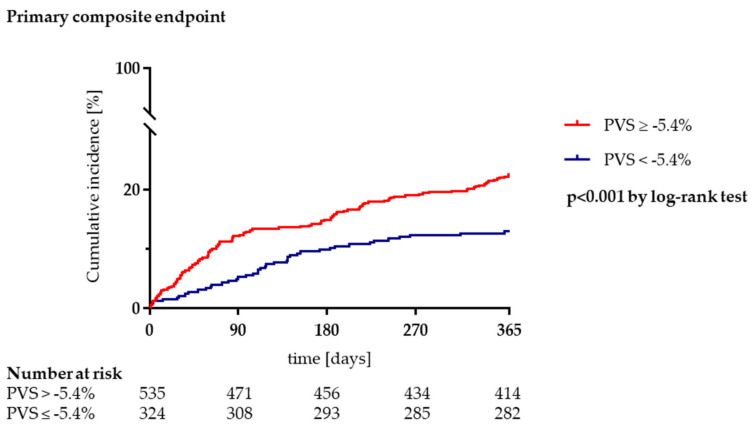
Elevated plasma volume status (PVS) is associated with the primary composite endpoint of all-cause mortality or heart failure hospitalization after transcatheter aortic valve implantation. Legend: Kaplan-Meier survival curves for the primary endpoint comparing patients with a PVS ≥ −5.4% (congestion) to patients with a PVS < −5.4% (no congestion).

**Figure 2:**
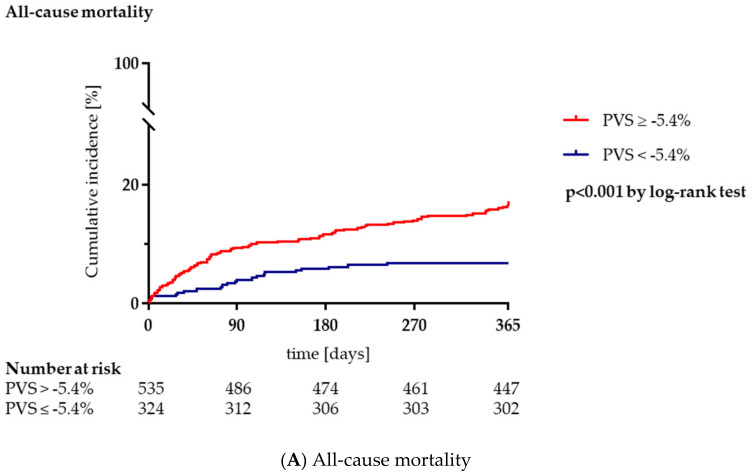

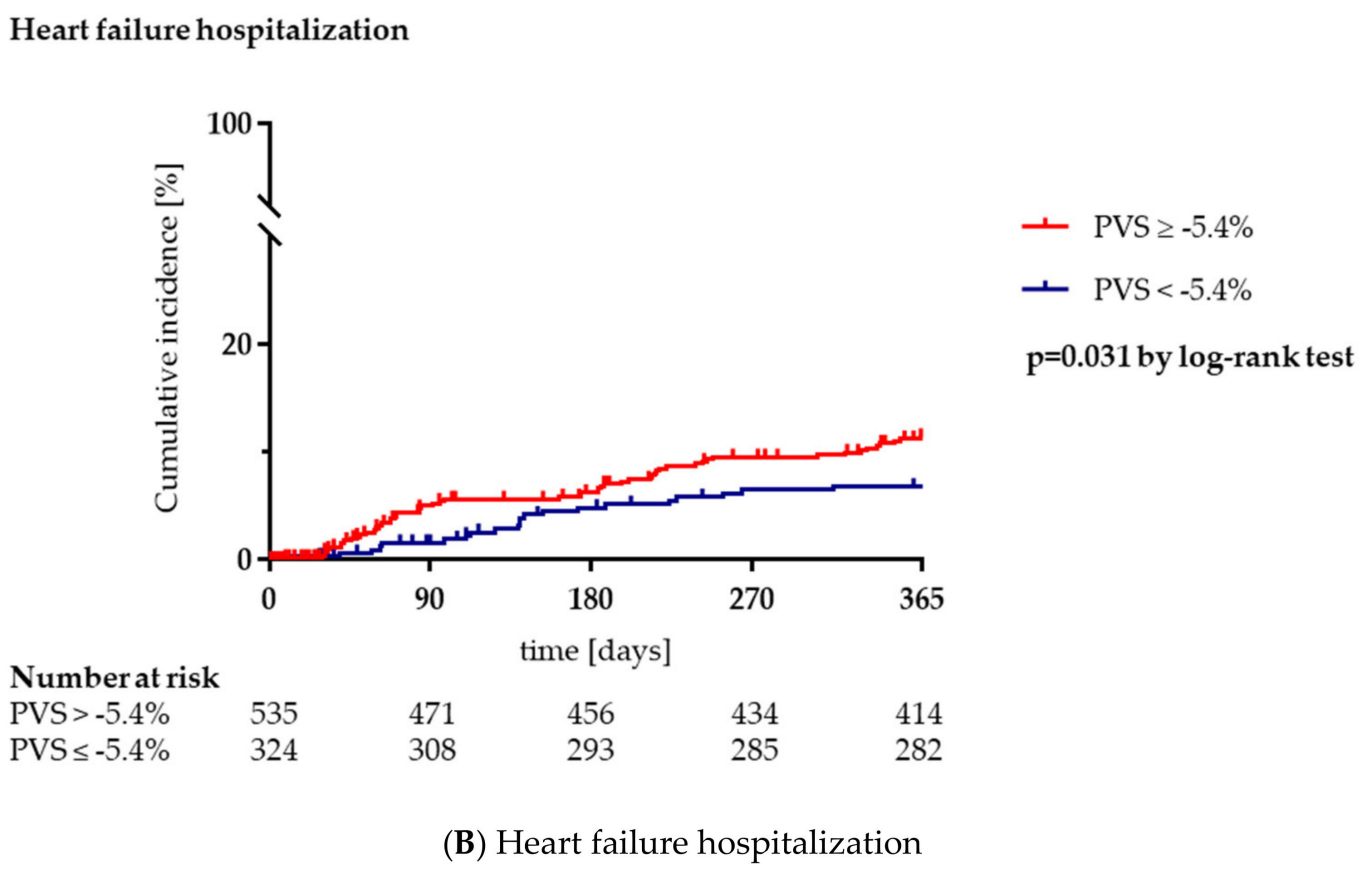
Elevated plasma volume status (PVS) is associated with adverse outcomes after transcatheter aortic valve implantation. (**A**) All-cause mortality. (**B**) Heart failure hospitalization. Legend: Kaplan-Meier survival curves for the secondary endpoints of all-cause mortality and heart failure hospitalization comparing patients with a PVS ≥ −5.4% (congestion) to patients with a PVS < −5.4% (no congestion).

The primary composite outcome (all-cause mortality or heart failure hospitalization) occurred in 121/535 patients (22.6%) in the PVS ≥ −5.4% group compared to 42/324 patients (13.0%) with a PVS < −5.4% (Table 1, Figure 1). Furthermore, the PVS ≥ −5.4% group had higher rates of both secondary endpoints of all-cause mortality (16.4% vs. 6.8%, *p* < 0.001) and heart failure hospitalization (10.5% vs. 6.5%, *p* = 0.031; Table 1, Figure 2A,B). In univariable Cox regression analysis, PVS was significantly associated with the primary endpoint (HR 1.99, 95%, CI 1.39–2.84, *p* < 0.001). After multivariable adjustment for other variables, PVS remained a significant predictor of the composite of all-cause mortality or heart failure hospitalization (HR 1.53, 95% CI 1.05–2.22, *p* = 0.026; Table 3).

## 4. Discussion

This study found that calculated PVS as a marker of hypervolemia was significantly associated with all-cause mortality and heart failure hospitalization in patients undergoing TAVI.

In our study, 62.3% of patients had a preprocedural PVS ≥ −5.4% reflecting relevant (subclinical) congestion. This relatively high incidence of hypervolemia as well as the ROC-derived cutoff for PVS of −5.4% in TAVI patients is consistent with previously published reports [14]. In another study using a PVS cut-off value of −4%, an elevated PVS was present in 59.6% of patients admitted for TAVI [15]. Compared to patients with PVS < −5.4%, patients with a PVS ≥ −5.4% in our study were older and had multiple co-morbidities. Again, this was consistent with previously published investigations, where elevated PVS was associated with increased comorbidities in patients undergoing TAVI [14,15,16,17]. The lower BMI in patients with a PVS ≥ −5.4% was most probably a result of malnutrition and frailty, which are associated with an increased risk of all-cause mortality in patients undergoing TAVI [16,18]. In our study, the median calculated PVS was −2.6% (−8.6–4.1%) which is in line with previously reported PVS in the context of TAVI [14]. Notably, PVS values in TAVI patients seem to be higher than in patients with chronic heart failure [8]. This finding indicates that patients with severe symptomatic AS may suffer from unrecognized hypervolemia and subclinical congestion to a greater extent compared to patients with chronic heart failure. This may be explained by the typical clinical profile of the TAVI population including advanced age, high prevalence of comorbidities, and suboptimal heart failure medication in elderly patients [19]. In our study, patients with a PVS ≥ −5.4% showed higher rates of diuretic therapy, while there was no significant difference in prognostic heart failure medication. This finding highlights the potential role of PVS as a tool to optimize medical treatment.

During follow-up, both mortality and heart failure hospitalization were significantly higher in patients with PVS ≥ −5.4% compared to patients with PVS < −5.4% (*p* < 0.001 and *p* = 0.031, respectively; Figure 2A,B). Consistently, other studies showed adverse outcomes in TAVI patients with elevated PVS. Maznyczka et al. reported that a PVS > 0% was linked to a two-fold risk for mortality as well as prolonged ICU and hospital stay [14]. Shimura et al. reported a significantly higher all-cause mortality and heart failure hospitalization rate in the high-PVS group than in the low-PVS group [17]. In addition, patients with high PVS and NYHA I/II had a worse prognosis than those with low PVS and NYHA III/IV. Adlbrecht et al. reported that patients with a high PVS did not only demonstrate a substantially impaired long-term survival during follow-up but were also at increased risk for a 30-day composite of all-cause mortality, stroke, life-threatening bleeding, acute kidney injury, coronary artery obstruction requiring intervention, major vascular complication and valve-related dysfunction requiring repeat procedure [15]. The prognostic significance of PVS was also previously reported in patients with heart failure. In an analysis of 3414 patients with heart failure with preserved ejection fraction, higher calculated estimates of PVS were independently associated with an elevated risk of long-term clinical outcomes, and particularly, heart failure hospitalization [20]. In an analysis of 186 patients who received a continuous-flow left ventricular assist device, high PVS was associated with higher mortality during follow-up [21].

Importantly, previous studies investigating calculated PVS have not reported the prevalence of anemia [14,15,17]. Based on WHO definitions, anemia (<12.0 g/dL in women; <13.0 g/dLs in men) was prevalent in 50.2% of the total population in our study (69.9% in the PVS ≥ −5.4% groups compared to 17.6% the PVS < −5.4% group, *p* < 0.001). This was mostly attributed to mild and moderate forms of anemia [22]. While anemia is known to be multifactorial in patients with severe AS, hemodilution due to congestion is likely to have had a significant impact on the high prevalence of anemia in patients with PVS ≥ −5.4% [23,24,25]. As was the case in previous studies, our analysis is unable to differentiate between “true” anemia and anemia due to hemodilution in patients with congestion. Thus, future studies should focus on the close relationship between anemia and PVS in order to correct for confounding effects.

It has been previously suggested that volume overload associated with elevated PVS might add to the pressure overload on the stiff, non-compliant ventricle in severe AS, leading to a higher risk of pulmonary and systemic edema, global hypoperfusion, and adverse outcomes after TAVI [14]. Thus, calculated PVS could be a simple tool to guide fluid management and medical treatment including diuretic therapy in patients with heart failure. Similarly, NT-proBNP has been previously proposed as a simple marker to guide therapy in the context of heart failure and TAVI [26,27]. Additionally, a number of cardiovascular biomarkers, such as high-sensitivity Troponin T, soluble ST2, and GDF-15 have also been studied in the context of TAVI and are associated with outcomes in patients undergoing TAVI [28]. Using PVS in patients with severe AS and optimizing heart failure treatment in addition to TAVI may result in more favorable outcomes in this vulnerable patient group. Prospective randomized trials are needed to study the utility of PVS as part of an integrated approach for improved risk stratification and management of TAVI patients.

There are several study limitations that have to be acknowledged. First, this is a single-center study which may limit the conclusions that can be drawn from the analysis. Second, in line with previous studies, hemoglobin/hematocrit and anemia were not included in the Cox regression model in order to minimize collinearity. However, comorbidities such as frailty, heart failure, and chronic kidney disease are known to be closely associated with hemoglobin concentrations, which may have significantly influenced PVS [29]. Third, as anemia and PVS are closely intertwined, this study is unable to differentiate between true anemia and anemia caused by hemodilution. Thus, anemia potentially remains a major confounding factor. Fourth, the relative low HR for PVS in the Cox regression analysis indicates that PVS should only be used as part of an integrative approach. Fifth, additional endpoints such as heart failure symptoms and physical capacity were not accounted for. Sixth, factors affecting PVS, such as blood transfusions and extensive heart failure therapy during a hospital stay, were not taken into consideration during the calculation of PVS. Finally, ePV in this study was not validated by the measured PV. However, ePV has been shown to correlate well with PV levels measured using gold-standard radioisotope assays [8,26].

## 5. Conclusions

Elevated PVS was an independent predictor of all-cause mortality and heart failure hospitalization within 1 year after TAVI. Future trials are necessary to determine the potential role of PVS as part of an integrative strategy towards improved risk stratification and therapeutic management of patients undergoing TAVI.

## Figures and Tables

**Table 1:** Baseline characteristics and heart failure medication.

	Total (*n* = 859)	PVS < −5.4% (*n* = 324)	PVS ≥ −5.4% (*n* = 535)	*p*-Value
Age (years)	81.9 (78.7–85.8)	81.0 (77.6–84.1)	82.8 (79.5–86.9)	<0.001
Female, *n* (%)	452 (52.6)	152 (46.9)	300 (56.1)	0.009
BMI (kg/m^2^)	26.2 (23.9–29.6)	28.9 (25.9–32.0)	25.0 (22.7–27.7)	<0.001
Atrial fibrillation, *n* (%)	373 (43.4)	134 (41.4)	239 (44.7)	0.342
CAD, *n* (%)	550 (64.0)	199 (61.4)	351 (65.6)	0.215
COPD, *n* (%)	94 (10.9)	31 (9.6)	63 (11.8)	0.315
CVD, *n* (%)	150 (17.5)	56 (17.3)	94 (17.6)	0.915
Diabetes mellitus, *n* (%)	258 (30.0)	96 (29.6)	162 (30.3)	0.840
Dyslipidemia, *n* (%)	431 (50.2)	177 (54.6)	254 (47.5)	0.042
Hypertension, *n* (%)	759 (88.4)	286 (88.3)	473 (88.4)	0.951
NYHA class III or IV, *n* (%)	612 (71.2)	219 (67.6)	393 (73.5)	0.067
PAD, *n* (%)	72 (8.4)	26 (8.0)	46 (8.6)	0.769
PAH, *n* (%)	115 (13.4)	46 (14.2)	69 (12.9)	0.588
Prev. cardiac surgery, *n* (%)	126 (14.7)	54 (16.7)	72 (13.5)	0.198
STS-Score (%)	3.7 (2.4–5.5)	2.9 (2.1–4.6)	4.2 (2.6–6.3)	<0.001
Hematocrit (%)	0.36 (0.32–0.39)	0.40 (0.38–0.42)	0.34 (0.31–0.36)	<0.001
Hemoglobin (g/dL)	12.4 (11.1–13.4)	13.6 (12.8–14.4)	11.5 (10.4–12.4)	<0.001
Anemia				
Mild	253 (29.5)	52 (16.0)	201 (37.6)	<0.001
Moderate	173 (20.1)	5 (1.5)	168 (31.4)	<0.001
Severe	5 (0.6)	0 (0)	5 (0.9)	0.081
Non-Anemia	428 (49.8)	267 (82.4)	161 (30.1)	<0.001
eGFR (mL/min/1.73 m^2^)	54 (40–67)	59 (48–70)	51 (37–63)	<0.001
NT-proBNP (pg/mL)	1461 (573–3462)	986 (445–2287)	2102 (704–4529)	<0.001
LVEF				
≥55%, *n* (%)	555 (64.6)	226 (69.8)	329 (61.5)	0.014
45–54%, *n* (%)	162 (18.9)	52 (16.0)	110 (20.6)	0.101
35–44%, *n* (%)	72 (8.4)	19 (5.9)	53 (9.9)	0.038
<35%, *n* (%)	70 (8.1)	27 (8.3)	43 (8.0)	0.878
AVA (cm^2^)	0.8 (0.6–0.9)	0.8 (0.6–0.9)	0.8 (0.6–0.9)	0.075
MPG (mmHg)	38 (29–50)	38 (30–49)	38 (29–50)	0.838
MR III-IV, *n* (%)	72 (8.4)	26 (8.0)	46 (8.6)	0.769
TR III-IV, *n* (%)	36 (4.2)	14 (4.3)	22 (4.1)	0.882
Calculated actual PV (mL)	2907 (2621–3264)	2951 (2693–3321)	2883 (2566–3235)	0.003
Calculated ideal PV (mL)	3000 (2613–3393)	3315 (3000–3783)	2800 (2457–3120)	<0.001
PVS (%)	−2.6 (−8.6–4.1)	−10.4 (−14.0–−7.5)	1.8 (−2.0–7.2)	<0.001
Heart failure medication				
ACE-I/ARB	714 (83.1)	275 (84.9)	439 (82.1)	0.285
Betablocker	639 (74.4)	235 (72.5)	404 (75.5)	0.332
MRA	108 (12.6)	35 (10.8)	73 (13.6)	0.223
Diuretics	598 (69.6)	211 (65.1)	387 (72.3)	0.026

Legend: ACE-I, angiotensin-converting enzyme inhibitor; ARB, angiotensin receptor blocker; AVA, aortic valve area; BMI, body mass index; CAD, coronary artery disease; COPD, chronic obstructive pulmonary disease; CVD, cerebrovascular disease; eGFR, estimated glomerular filtration rate; LVEF, left ventricular ejection fraction; MPG, mean pressure gradient; MR, mitral regurgitation; MRA, mineralocorticoid receptor antagonist; NT-proBNP, N-terminal Pro-B-Type Natriuretic Peptide; NYHA, New York Heart Association; PAD, peripheral artery disease; PAH, pulmonary arterial hypertension; Prev., previous; PV, plasma volume; PVS, plasma volume status; STS, Society of Thoracic Surgeons; TR, tricuspid regurgitation. Values are presented as median (interquartile range) or counts (percentages).

**Table 2:** Procedural variables and outcomes.

	Total (*n* = 859)	PVS < −5.4% (*n* = 324)	PVS ≥ −5.4% (*n* = 535)	*p*-Value
Valve type				0.146
Self-expanding, *n* (%)	433 (50.4)	153 (47.2)	280 (52.3)	
Balloon-expandable, *n* (%)	426 (49.6)	171 (52.8)	255 (47.7)	
Procedural duration (min)	50 (40–63)	49 (40–61)	49 (40–65)	0.302
Contrast medium (mL)	84 (70–105)	85 (70–107)	80 (68–103)	0.219
VARC-3				
New permanent pacemaker, *n* (%)	101 (11.8)	43 (13.3)	58 (10.8)	0.284
Myocardial infarction, *n* (%)	3 (0.3)	1 (0.3)	2 (0.4)	>0.999
AKIN stage 3/4, *n* (%)	9 (1.0)	2 (0.6)	7 (1.3)	0.496
Type 3 (life-threatening) bleeding, *n* (%)	21 (2.4)	4 (1.2)	17 (3.2)	0.108
Stroke with disability, *n* (%)	3 (0.3)	1 (0.3)	2 (0.4)	>0.999
Primary composite outcome, *n* (%)	163 (19.0)	42 (13.0)	121 (22.6)	<0.001
All-cause mortality, *n* (%)	110 (12.8)	22 (6.8)	88 (16.4)	<0.001
Heart failure hospitalization, *n* (%)	77 (9.0)	21 (6.5)	56 (10.5)	0.048

Legend: AKIN, Acute Kidney Injury Network; PVS, plasma volume status; VARC-3, Valve Academic Research Consortium-3. Values are presented as median (interquartile range) or counts (percentages).

**Table 3:** Cox regression analysis for the primary composite endpoint of all-cause mortality or heart failure hospitalization.

Variable	HR (95% CI)	*p*-Value	HR (95% CI)	*p*-Value
PVS ≥ −5.4%	1.99 (1.39–2.84)	<0.001	1.53 (1.05–2.22)	0.026
Atrial fibrillation	1.70 (1.25–2.31)	<0.001		
Age > median (81.9 years)	1.27 (0.93–1.73)	0.132		
BMI > median (26.2 kg/m^2^)	0.66 (0.48–0.90)	0.008		
STS score > median (3.7%)	2.23 (1.61–3.08)	<0.001	1.67 (1.17–2.38)	0.005
COPD	1.58 (1.03–2.42)	0.036	1.54 (0.99–2.39)	0.056
NT-proBNP > median (1461 pg/mL)	1.94 (1.41–2.67)	<0.001	1.48 (1.06–2.09)	0.023
eGFR < 60 mL/min/1.73 cm^2^	1.85 (1.29–2.65)	<0.001		
LVEF < 55%	1.73 (1.27–2.35)	<0.001		
TR III-IV	3.37 (2.04–5.57)	<0.001	2.87 (1.72–4.80)	<0.001
MR III-IV	2.36 (1.55–3.59)	<0.001		
PAH	1.55 (1.04–2.30)	0.030		
AKIN stage 3/4	10.10 (4.72–21.70)	<0.001	8.50 (3.91–18.51)	<0.001
Myocardial infarction	5.11 (1.27–20.60)	0.022	6.12 (1.47–25.40)	0.013
Stroke with disability	7.73 (1.92–31.20)	0.004		
Type 3 (life-threatening) bleeding	4.60 (2.55–8.28)	<0.001	6.34 (3.50–11.49)	<0.001

Legend: AKIN, Acute Kidney Injury Network; BMI, body mass index; COPD, chronic obstructive pulmonary disease; eGFR, estimated glomerular filtration rate; LVEF, left ventricular ejection fraction; MR, mitral regurgitation; NT-proBNP, N-terminal Pro-B-Type Natriuretic Peptide; PAH, pulmonary arterial hypertension; PVS, plasma volume status; STS, Society of Thoracic Surgeons; TR, tricuspid regurgitation. Results are presented as adjusted hazard ratios (HR) with 95% confidence intervals (CI).

## Data Availability

The datasets for this study will not be made available to other researchers due to data protection reasons. However, calculation of PVS is simple and easily reproducible, and we encourage scientists to validate our findings in other TAVI cohorts.
